# Sodium channel **β**1 subunits participate in regulated intramembrane proteolysis-excitation coupling

**DOI:** 10.1172/jci.insight.141776

**Published:** 2021-02-08

**Authors:** Alexandra A. Bouza, Nnamdi Edokobi, Samantha L. Hodges, Alexa M. Pinsky, James Offord, Lin Piao, Yan-Ting Zhao, Anatoli N. Lopatin, Luis F. Lopez-Santiago, Lori L. Isom

**Affiliations:** 1 Department of Pharmacology, University of Michigan Medical School, Ann Arbor, Michigan, USA.; 2 Department of Molecular & Integrative Physiology, University of Michigan Medical School, Ann Arbor, Michigan, USA.; 3 Department of Neurology, University of Michigan Medical School, Ann Arbor, Michigan, USA.

**Keywords:** Cardiology, Cell Biology, Cell migration/adhesion, Sodium channels, Transcription

## Abstract

Loss-of-function (LOF) variants in *SCN1B,* encoding voltage-gated sodium channel β1 subunits, are linked to human diseases with high risk of sudden death, including developmental and epileptic encephalopathy and cardiac arrhythmia. β1 Subunits modulate the cell-surface localization, gating, and kinetics of sodium channel pore-forming α subunits. They also participate in cell-cell and cell-matrix adhesion, resulting in intracellular signal transduction, promotion of cell migration, calcium handling, and regulation of cell morphology. Here, we investigated regulated intramembrane proteolysis (RIP) of β1 by BACE1 and γ-secretase and show that β1 subunits are substrates for sequential RIP by BACE1 and γ-secretase, resulting in the generation of a soluble intracellular domain (ICD) that is translocated to the nucleus. Using RNA sequencing, we identified a subset of genes that are downregulated by β1-ICD overexpression in heterologous cells but upregulated in *Scn1b*-null cardiac tissue, which lacks β1-ICD signaling, suggesting that the β1-ICD may normally function as a molecular brake on gene transcription in vivo. We propose that human disease variants resulting in *SCN1B* LOF cause transcriptional dysregulation that contributes to altered excitability. Moreover, these results provide important insights into the mechanism of *SCN1B*-linked channelopathies, adding RIP-excitation coupling to the multifunctionality of sodium channel β1 subunits.

## Graphical abstract

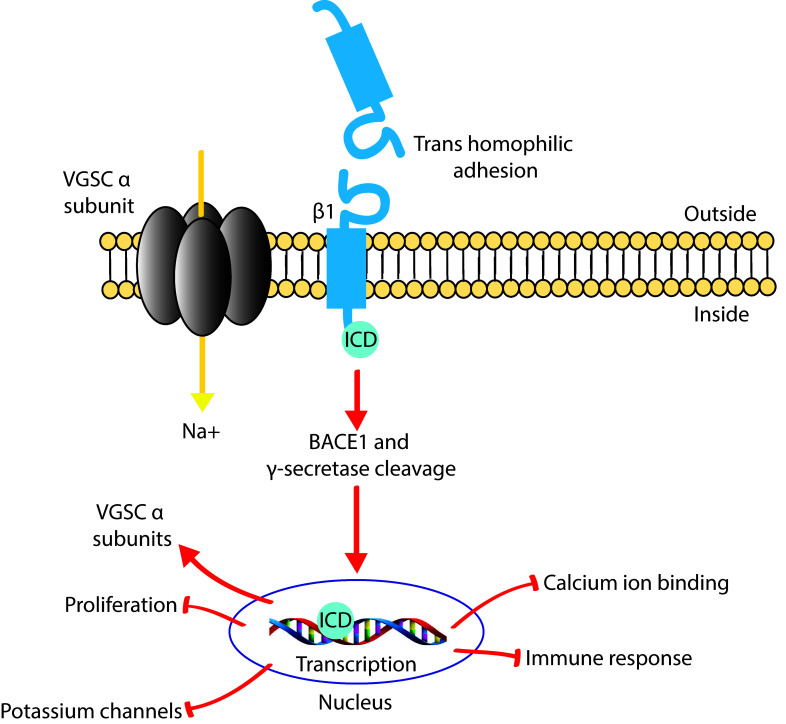

## Introduction

Loss-of-function (LOF) variants in *SCN1B,* encoding (VGSC) β1 subunits, are linked to human diseases that carry a high risk of sudden death, including developmental and epileptic encephalopathy type 52 (DEE52, OMIM 617350), Brugada syndrome 5 (OMIM #612838), and atrial fibrillation, familial, 13 (OMIM #615377). β1 Subunits are type 1 transmembrane proteins containing a single extracellular V-type Ig domain, making them part of the Ig superfamily of cell adhesion molecules (CAMs) (1, 2). β1 Subunits are multifunctional proteins. In addition to their canonical roles in modulating the cell-surface localization, gating, and kinetics of sodium channel pore-forming α subunits (3, 4), β1 subunits modulate potassium currents and participate in cell-cell and cell-matrix adhesion as CAMs (5–8). *Scn1b*-null mice, which model DEE52, have cell type–specific alterations in sodium current (9–13), multiple deficits in neuronal migration and pathfinding in the cerebellum (14), fewer nodes of Ranvier in the optic nerve (15), aberrant neuronal pathfinding and fasciculation in the corticospinal tract (16), delayed maturation of GABAergic signaling in the brain (17), abnormal formation of cardiac intercalated discs (18), and altered calcium signaling in cardiac ventricular myocytes (12). Finally, sodium channel β1 subunits are essential for normal development: *Scn1b*-null mice have severe seizures, ataxia, and cardiac arrhythmia, with 100% mortality by postnatal day 21 (10, 15).

**Figure 1:**
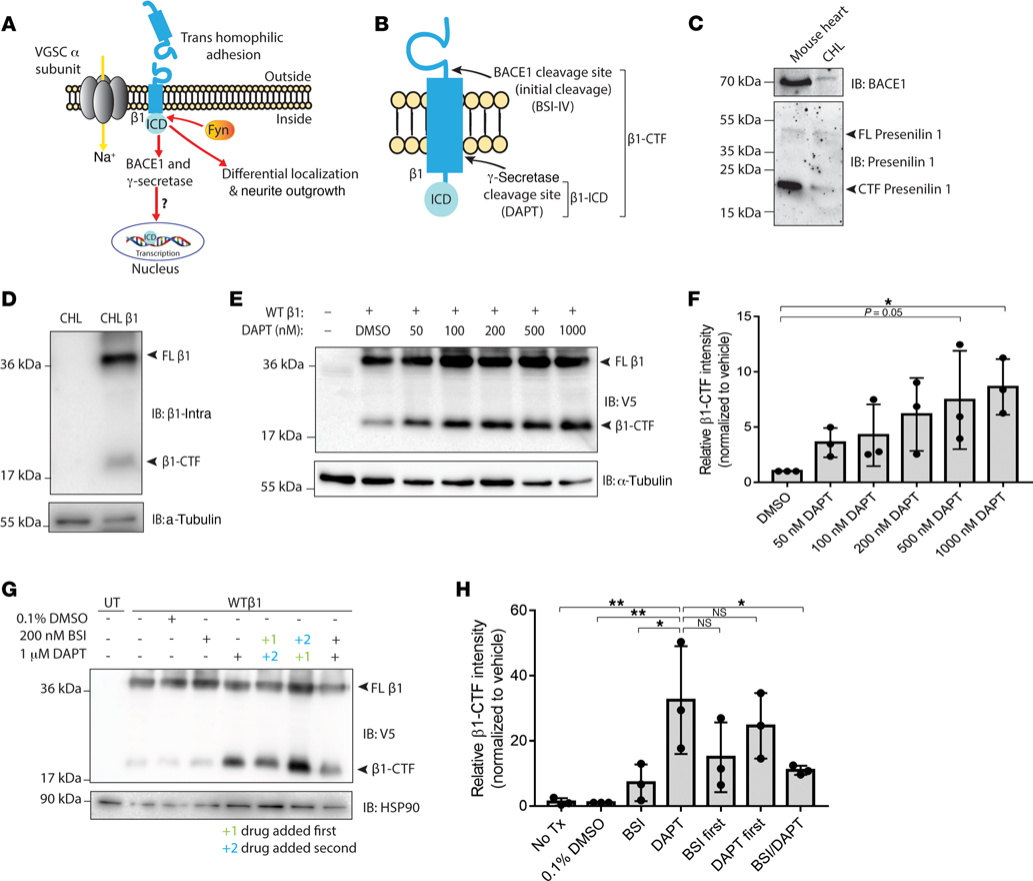
β1 subunits are substrates for BACE1 and γ-secretase intramembrane cleavage. (**A**) Cartoon diagram of the proposed β1-mediated signal transduction cascade. (**B**) Schematic of β1 with BACE1 and γ-secretase cleavage sites. (**C**) Chinese hamster lung (CHL) cells stably expressing WT β1-V5 also endogenously express BACE1 and presenilin-1, the catalytic subunit of γ-secretase. (**D**) WT β1-V5 is cleaved by BACE1, and the β1-C-terminal fragment (β1-CTF) is found in the membrane fraction. (**E**) Treatment with γ-secretase inhibitor, DAPT, leads to a concentration-dependent accumulation of β1-CTF. (**F**) Quantification of **E**. Protein levels were normalized to the loading control and reported as fold change respective to the vehicle-treated group. Significance (*P* value less than 0.05) was determined using a 1-way ANOVA between each treatment and the negative control (vehicle treatment). (**G**) Scheduled treatments with DAPT and β-secretase inhibitor IV inhibit formation of respective cleavage products in a manner consistent with sequential cleavage. (**H**) Quantification of **G**. Protein levels were normalized to the loading control and reported as fold change respective to the vehicle-treated group. Significance (*P* value less than 0.05) was determined using a 1-way ANOVA between each treatment and the positive control (DAPT treatment alone). Data represent mean ± SEM. For each experiment, *n* = 3. See complete unedited blots in the supplemental material.

Previous work by others showed that β1 subunits undergo regulated intramembrane proteolysis (RIP) through sequential cleavage by the β-site amyloid precursor protein (APP) cleaving enzyme-1 (BACE1) and γ-secretase (19). Initial cleavage by BACE1 sheds the β1 extracellular Ig domain, which our laboratory previously showed functions as a ligand for cell adhesion, and leaves the β1-C-terminal fragment (β1-CTF) in the membrane (20, 21). Cleavage by BACE1 was reported to be the rate-limiting step in β1 RIP. γ-Secretase subsequently cleaves the remaining β1-CTF in the lumen of the membrane, generating a soluble intracellular domain (β1-ICD) (Figure 1B) (19).

Although typically studied as neuronal enzymes, BACE1 and γ-secretase are expressed ubiquitously throughout the body and have been shown to play important roles in other tissues. For example, in cardiac myocytes, KCNE1, which assembles with KCNQ1 channels to generate delayed-rectifier potassium current, is a BACE1 substrate (22, 23). Atrial cardiomyocytes isolated from *Bace1-*null mice show a decrease in total steady-state potassium current (22). Presenilins, the catalytic component of the γ-secretase complex, have been implicated in degradation of the ryanodine receptor in cardiomyocytes (24). BACE1 and γ-secretase also play roles in cancer. For example, inhibitors of γ-secretase inhibit growth of human glioblastoma as well as human lung adenocarcinoma tumors xenografted into nude mice (23–27).

Evidence from other BACE1 and γ-secretase substrates suggests that ICDs generated by RIP are translocated to the cell nucleus where they modulate transcription (28, 29). Based on this evidence, we proposed that the β1-ICD may participate in transcriptional regulation in vivo and that the absence of β1 RIP and downstream signaling may contribute to disease mechanisms in patients with LOF *SCN1B* variants. Thus, the goal of this study was to test the hypothesis that the β1-ICD couples membrane excitability, as a sodium channel subunit, to transcriptional regulation (RIP-excitation coupling). Using an unbiased RNA sequencing (RNA-Seq) approach, we identified multiple gene pathways that are downregulated by β1-ICD overexpression in heterologous cells. As a result of our previous work showing the important role of *Scn1b* in cardiac physiology, we also performed RNA-Seq to examine gene expression profiling in mouse cardiac ventricle isolated from P10 *Scn1b* WT versus *Scn1b-*null animals, in which the β1-ICD signaling pathway is deleted. Overall, we found the gene groups that were downregulated by β1-ICD overexpression in heterologous cells were upregulated in the *Scn1b*-null cardiac ventricle, suggesting that the β1-ICD may normally act as a molecular brake on gene expression in heart. We showed that the observed transcriptional upregulation of potassium channel gene expression translates to increased potassium currents in *Scn1b*-null cardiac myocytes. Finally, we showed that calcium current is decreased in *Scn1b-*null ventricular myocytes, consistent with alterations in calcium ion binding proteins (CBPs) and calcium channel modulatory proteins identified by RNA-Seq experiments. Taken together, our work identifies a sodium channel β1-mediated signal transduction cascade in heart with physiological implications for the regulation of normal development as well as pathology. In addition, the absence of β1 RIP and downstream signaling, as modeled by *Scn1b-*null mice, may contribute to cardiac disease mechanisms in patients with *SCN1B* LOF variants.

## Results

### β1 is sequentially cleaved by BACE1 and γ-secretase in vitro.

We used stably transfected Chinese hamster lung (CHL) fibroblasts to confirm previous results identifying β1 as a substrate for RIP by BACE1 and γ-secretase, as well as to establish a model system to study downstream signaling from β1 cleavage (Figure 1, A and B) (19). CHL cells are optimal for this work because they do not express endogenous sodium channel β1 subunit mRNA (30), but do express low levels of both BACE1 and γ-secretase (Figure 1C). We generated a bicistronic, full-length WT sodium channel β1 subunit cDNA expression vector containing a C-terminal V5 epitope tag, a cleaving 2A sequence, and enhanced GFP (eGFP) to establish a stable β1-V5-2AeGFP-CHL cell line. Western blot analysis of cell lysates revealed an immunoreactive band at approximately 37 kDa, the expected molecular weight (MW) of full-length β1. An additional band was present at approximately 20 kDa, consistent with the previously identified apparent MW of the β1-CTF that remains in the membrane following initial cleavage by BACE1 (Figure 1D) (19). To determine if the approximately 20 kDa fragment was the β1-CTF, β1-V5-2AeGFP-CHL cells were treated with vehicle (0.1% DMSO) or increasing concentrations (50–1000 nM) of the γ-secretase inhibitor (DAPT) (19, 29). If the approximately 20 kDa fragment was indeed the β1-CTF, DAPT would block the second cleavage event by γ-secretase, leading to an accumulation of the intermediary cleavage product produced by BACE1, β1-CTF, in the membrane. DAPT treatment resulted in a concentration-dependent accumulation of the 20 kDa fragment, suggesting that this band represented β1-CTF, which would have been subsequently processed by γ-secretase in the absence of drug (Figure 1, E and F).

To determine if initial β1 cleavage was the result of BACE1 activity, rather than activity of another protease, e.g., an α-secretase, β1-V5-2AeGFP-CHL cells were treated with vehicle (0.1% DMSO), 1000 nM DAPT, or 200 nM β-secretase inhibitor IV (BSI) (29), varying the order of addition. Treatment of cells with 200 nM BSI alone did not alter the β1-CTF level, as assessed by Western blot. Treatment with DAPT to accumulate the β1-CTF was required to detect differences in the amount of β1-CTF generated. Coadministration of BSI plus DAPT resulted in a significant decrease in the β1-CTF level generated in comparison with DAPT treatment alone. Treatment with DAPT for 7 hours, to allow for β1-CTF accumulation prior to BSI treatment, did not change the amount of β1-CTF generated because BACE1 cleavage is the rate-limiting step. Inhibiting the initial cleavage after blocking the second γ-secretase–mediated cleavage event had little to no effect, as initial cleavage had already occurred. Taken together, these results suggest that RIP of β1 occurs sequentially, with initial cleavage by BACE1 (Figure 1, G and H). The data presented in Figure 1 strengthen previous evidence showing that β1 is a substrate for sequential cleavage by BACE1, which generates the β1-CTF, followed by γ-secretase, generating the β1-ICD.

### The β1-ICD localizes to the nucleus.

**Figure 2:**
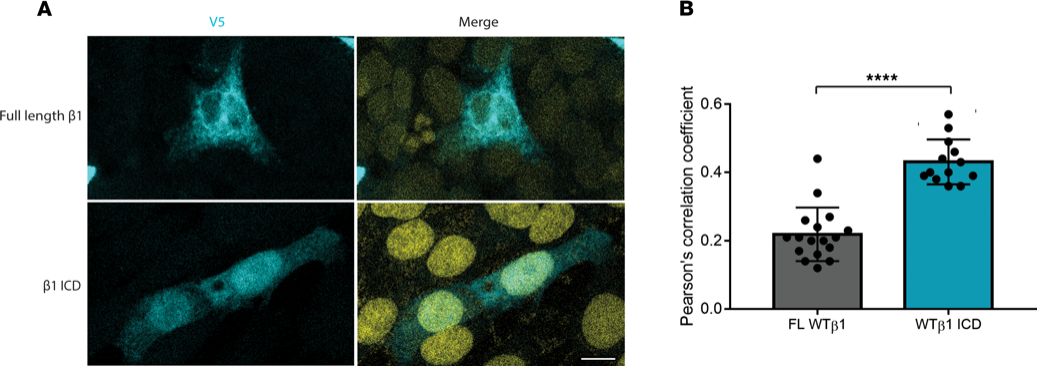
β1-ICD-V5 localizes to the nucleus. (**A**) Full-length WT β1-V5 shows little to no nuclear localization, as determined by staining for the V5-epitope tag and DAPI. Strong colocalization is observed between staining for the V5-epitope tag of the β1-ICD and the nucleus (DAPI, yellow). (**B**) Quantification of intensity of V5 and DAPI staining across the transfected cell. Averaged data from 13–17 cells per condition are shown from 3 independent transfections. Data represent mean ± SD. Statistical significance was determined using Student’s *t* test.

To determine if the β1-ICD localizes to the nucleus, similar to other substrates of intramembrane sequential BACE1 and γ-secretase cleavage, we cloned and transiently expressed WT β1-V5 or β1-ICD-V5 in CHL cells (28). Our previous work showed that addition of the in-frame C-terminal V5-epitope tag had no effect on β1 function compared with untagged β1 (31). Immunofluorescence staining with anti-V5 showed that, unlike full-length β1-V5, for which there was little nuclear staining, approximately 50% of the expressed β1-ICD-V5 localized to the nucleus of CHL cells, as quantified by Pearson’s correlation coefficient for colocalization with DAPI (Figure 2, A and B).

### β1-ICD overexpression in heterologous cells leads to differential expression of VGSC genes.

**Figure 3:**
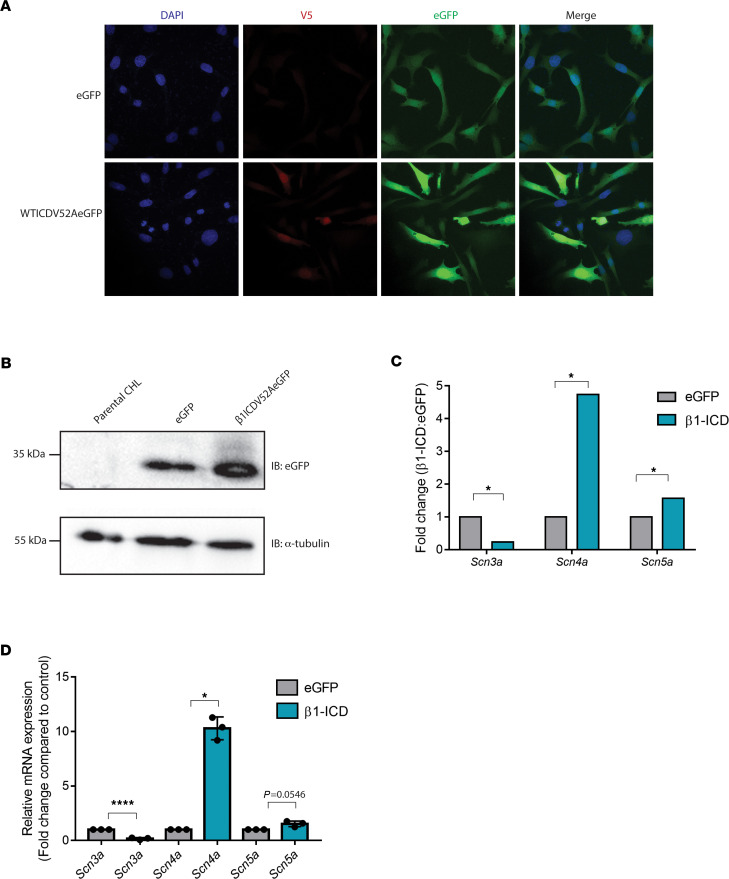
β1-intracellular domain expression alters voltage-gated sodium channel gene expression. (**A**) Immunocytochemistry for V5 for the enhanced GFP (eGFP) stable line and WT β1-ICD-V5-2A-eGFP (positive control) stable line. DAPI is shown in blue, eGFP in green, and V5 in red. (**B**) Immunoblot (anti-GFP) of CHL cells stably overexpressing eGFP only or WT β1-intracellular domain (β1-ICD0 with a V5 epitope tag and a 2A-eGFP sequence on the 3′ end. (**C**) RNA-Seq identifies VGSC genes are differentially expressed in presence of β1-ICD compared with control; *n* = 4. Gene transcripts were considered differentially expressed if they had a fold-change greater than or equal to 1.5 and a false discovery rate less than 0.05. (**D**) qPCR confirms some voltage-gated sodium channel (VGSC) genes are differentially expressed in the presence of β1-ICD compared with control; *n* = 3. Data represent mean ± SEM. Statistical significance was determined using Student’s *t* test.

After identifying the β1-ICD in the nucleus, we wanted to determine whether the β1-ICD could modulate transcription. To investigate this problem, we generated CHL cell lines, which stably overexpressed either eGFP or β1-ICD-V52A-eGFP (Figure 3, A and B). Paired-end RNA-Seq was performed on each cell line as fee-for-service by the University of Michigan Sequencing Core. Data were normalized, and differential expression analysis was performed with DESeq2 as fee-for-service by the University of Michigan Bioinformatics Core. Samples grouped according to genotype by principal component analysis (PCA) (Supplemental Figure 1A; supplemental material available online with this article; https://doi.org/10.1172/jci.insight.141776DS1). A total of 1396 genes were found to be differentially expressed in the β1-ICD line compared with the eGFP-only control line (Supplemental Figure 1C). Notably, of the genes identified using this unbiased approach, 3 VGSC α subunit genes were identified as differentially expressed: *Scn3a*, encoding Nav1.3, was downregulated in the β1-ICD overexpressing line compared with the control line, whereas both *Scn4a* and *Scn5a*, encoding Nav1.4 and Nav1.5, respectively, were upregulated compared with the control line (Figure 3C). Reverse transcription quantitative PCR (RT-qPCR) experiments confirmed these alterations in VGSC gene expression in the presence of the β1-ICD (Figure 3D).

To determine whether β1-ICD overexpression in heterologous cells was sufficient to drive changes in sodium current, either by direct interaction with the channel complex or by inducing changes in endogenous sodium channel gene expression, we overexpressed β1-ICD-V5-2A-eGFP or eGFP in human embryonic kidney (HEK) cells using transient transfection and recorded sodium currents using whole-cell voltage clamp approximately 24 hours later (Supplemental Figure 2A). Four independent experiments were performed per condition. No significant differences in sodium current density were observed in cells expressing the β1-ICD compared with control. We next transiently transfected cells that stably expressed sodium current, HEK-hNa_v_1.5 cells, with eGFP (negative control), WT β1-V5-2A-eGFP (positive control), or β1-ICD-V5-2A-eGFP to determine whether β1-ICD expression could change sodium current density. eGFP-positive cells were analyzed by whole-cell patch clamp 24 hours after transfection. Four independent experiments were performed per condition. β1-ICD coexpression with hNa_v_1.5 did not significantly change sodium current density or the voltage dependence of sodium current activation or inactivation compared with eGFP alone. Taken together, these results suggest that the combined β1-ICD–driven up- and downregulation of sodium channel α subunit gene expression may not be sufficient to change whole-cell sodium current density in heterologous cells (Supplemental Figure 2, B–E). Nevertheless, our previous in vivo work, repeated for this study and shown in Supplemental Figure 5, demonstrating that *Scn1b* deletion results in upregulation of *Scn3a* and *Scn5a/*Nav1.5 expression and increased sodium current density in cardiac myocytes is consistent with the idea that the β1-ICD regulates these genes (10, 12). However, the magnitude and direction of these changes in expression (up or down) may be specific for cell type and/or developmentally regulated.

In addition to their nuclear functions, some ICDs play local roles at their site of cleavage (32). To test whether acute application of the β1-ICD could modulate sodium current directly, we applied a synthetic β1-ICD peptide (β1_183–218_) through the patch pipet during whole-cell voltage clamp recording of HEK-hNav1.5 cells. No significant differences in sodium current density or in the voltage dependence of activation or inactivation were observed with the addition of the peptide (Supplemental Tables 1 and 2).

### Complex, concomitant patterning of gene expression changes.

**Figure 4:**
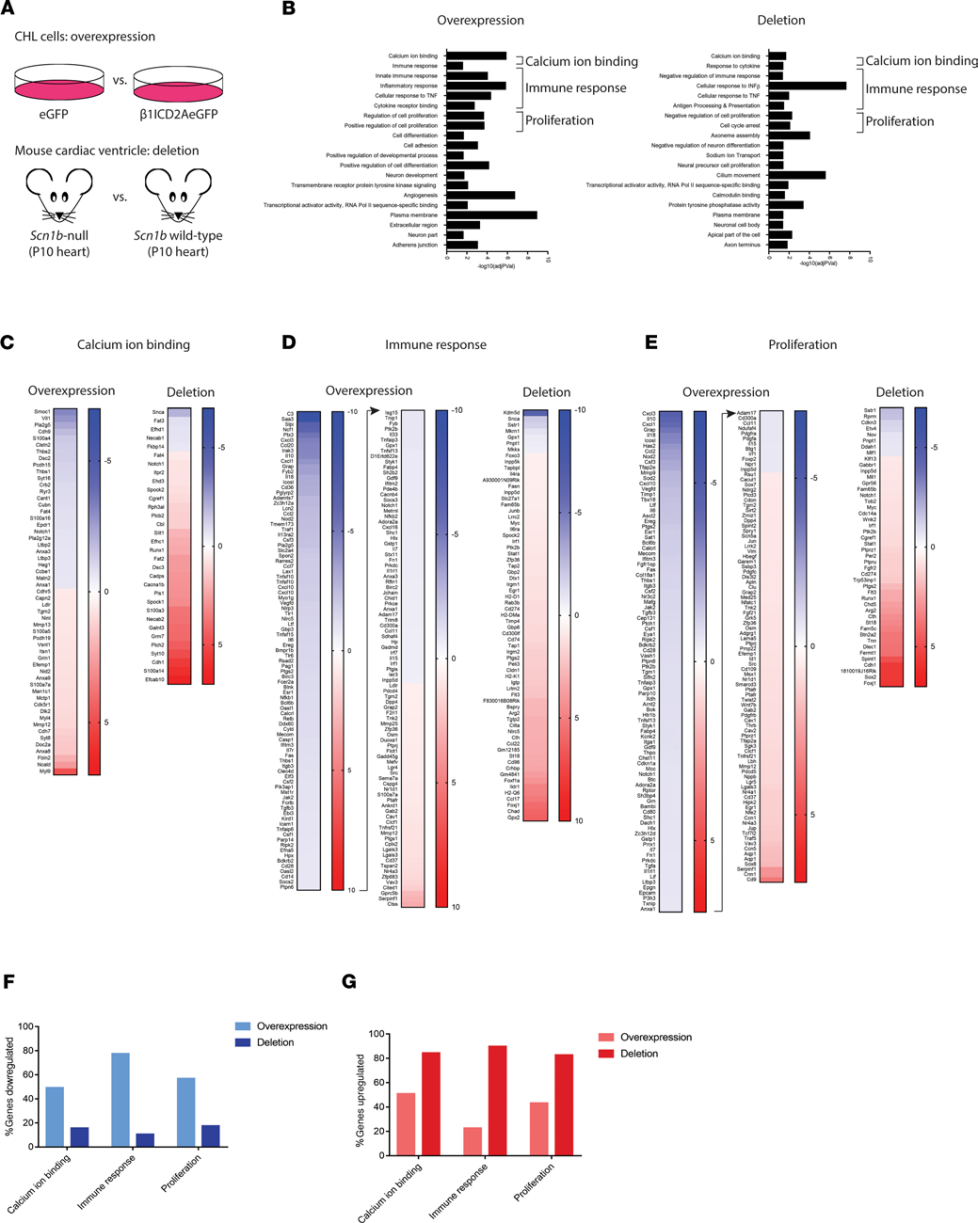
β1-ICD modulates gene transcription in vitro and in vivo. (**A**) Experimental design (*n* = 4 samples for each condition, all run in 1 RNA-Seq experiment). (**B**) Gene ontology (GO) groups overrepresented in analysis from CHL cells overexpressing the β1-ICD (left) and *Scn1b*-null cardiac ventricle (right). (**C–E**) Heat maps depicting genes altered in each RNA-Seq related to calcium ion binding (**C**), the immune response (**D**), and proliferation (**E**). (**F**) Percent of genes downregulated in each data set for calcium ion binding, immune response, and proliferation GO groups. (**G**) Percent of genes upregulated in each data set for calcium ion binding, immune response, and proliferation GO groups.

Gene ontology (GO) analysis revealed groups of genes that were changed by overexpression of the β1-ICD as measured by RNA-Seq (Figure 4A). The primary sets of genes differentially expressed included, but were not limited to, those involved in cell adhesion, the immune response, cellular proliferation, and calcium ion binding. (Figure 4B, left). To determine whether the expression of any of these sets of genes was also modulated in an excitable tissue that is known to normally express *Scn1b*, and for which *Scn1b* LOF is critical to disease mechanisms, we performed a second RNA-Seq experiment from P10 *Scn1b* WT and -null mouse cardiac ventricle (Figure 4A and Supplemental Figure 3). We chose P10 because this developmental time point is prior to disease onset in *Scn1b*-null mice, and thus uncomplicated by possible secondary effects of epilepsy (17). Paired-end RNA-Seq, normalization of data, and differential expression analysis with DESeq2 were performed as described above. Samples grouped according to genotype by PCA (Supplemental Figure 1B). A total of 696 genes were found to be differentially expressed between *Scn1b* WT and -null tissues (Supplemental Figure 1D). Although some of these changes in gene expression may be compensatory to deletion of the full-length β1 protein, rather than solely to the absence of the β1-ICD, we hypothesized that those which changed in a manner consistent with genes altered by β1-ICD overexpression may result from the loss of this signaling pathway. GO analysis revealed many similar groups of differentially expressed genes as in the CHL cell experiment, including genes in the immune response, proliferation, and calcium ion binding pathways (Figure 4B, right). Notably, in heterologous cells where the β1-ICD was overexpressed, most of these gene groups were downregulated. In contrast, where the β1-ICD was deleted (*Scn1b*-null cardiac tissue), these same gene groups were generally upregulated (Figure 4, C–G). Taken together, these data suggest that the β1-ICD may normally participate in gene repression in vivo. In contrast to our previous work showing increased *Scn3a* and *Scn5a* expression at P14-17 in *Scn1b-*null hearts (10, 12), the present RNA-Seq results showed no changes in sodium channel gene expression at P10. Thus, *Scn1b* deletion may lead to developmentally regulated changes in sodium channel α subunit expression in heart (10, 12). We tested this hypothesis using RT-qPCR in WT and *Scn1b*-null cardiac ventricle isolated from P16-17 animals. These results showed increased *Scn3a* and *Scn5a* expression, with decreased *Scn4a* expression. *Scn1b* deletion was also confirmed using RT-qPCR as a control (Supplemental Figure 4, A–D). To ask whether the observed changes in sodium channel gene transcription resulted in detectable changes in sodium current at P16-P17, we performed whole-cell voltage clamp analysis of acutely isolated cardiac ventricular myocytes. Consistent with previous results (10) and changes in gene expression described above, sodium current density was increased in P16-17 *Scn1b*-null cardiac ventricular myocytes compared with WT (Supplemental Figure 5).

### Potassium currents are increased in Scn1b-null ventricular cardiac myocytes.

**Figure 5:**
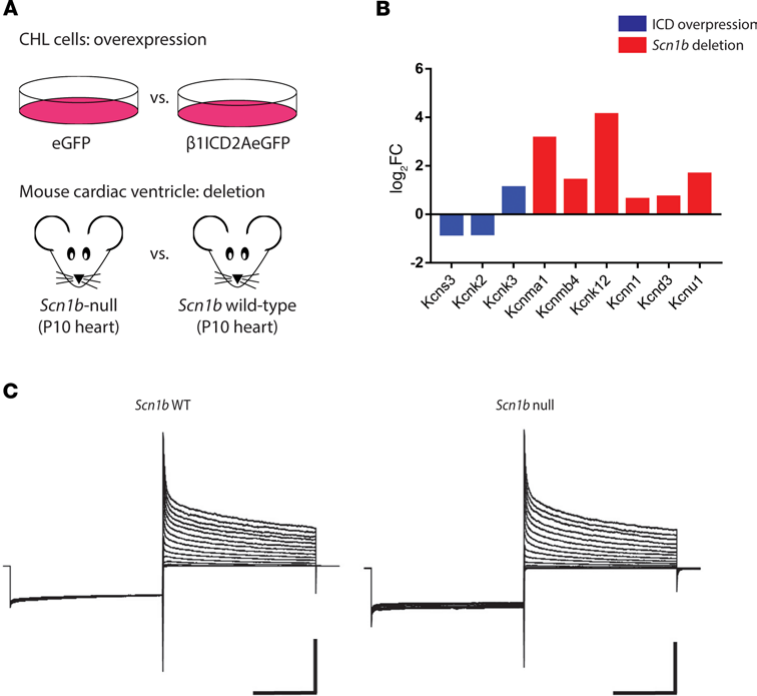
β1-ICD regulates potassium channel gene expression and potassium currents in cardiac ventricular myocytes. (**A**) Experimental design of RNA-Seq experiments from CHL cells stably overexpressing the β1-ICD and from P10 *Scn1b* WT or *Scn1b*-null mouse cardiac ventricle. (**B**) RNA-Seq showed that β1-ICD expression downregulates potassium channel genes, whereas *Scn1b*-null mice show upregulated potassium channel gene expression in cardiac ventricle. (**C**) Representative potassium currents recorded from ventricular myocytes obtained from WT and *Scn1b*-null mice. To assess the I-V relationship, 5-second pulses were applied in +10 mV increments from –70 mV to +70 mV, following a 5-second prepulse to –120 mV from –70 mV holding potential. Scale bars: 5 nA and 2 seconds.

β1-ICD overexpression in CHL cells resulted in changes in potassium channel gene expression compared with controls: downregulation of *Kcns3* (Kv9.3) and *Kcnk2* (TREK-1) and upregulation of *Kcnk3* (TASK-1). In contrast, a set of potassium channel genes, *Kcnma1* (KCa1.1 or BK)*, Kcnmb4* (BK-Beta4)*, Kcnk12* (THIK-2)*, Kcnn1* (KCa2.1 or SK)*, Kcnd3* (Kv4.3)*,* and *Kcnu1* (Slo3), were upregulated in *Scn1b-*null cardiac ventricular tissue, in which the β1-ICD signaling pathway is deleted (Figure 5, A and B). We performed RT-qPCR in P16-P17 WT and *Scn1b*-null cardiac ventricle to investigate whether *Kcnd3, Kcne1,* or *Kcnq1* potassium channel gene expression changed with development. Consistent with RNA-Seq results for P10 animals, *Kcnd3* and *Kcnq1* were upregulated, whereas *Kcne1* was downregulated at P16-17 (Supplemental Figure 4, F–H).

**Figure 6:**
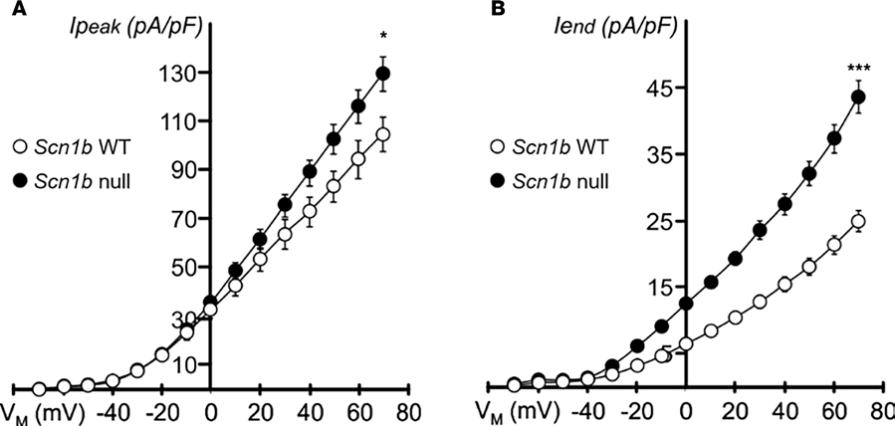
Comparison of current density and voltage dependence of inactivation of peak and end potassium currents. (**A and B**) *Scn1b* deletion results in increased peak (I_peak_) (**A**) and end (I_end_) (**B**) potassium current densities at depolarized potentials. *P ≤ 0.05, ***P ≤ 0.001 by Student’s *t* test (assuming equal variances). (**A**) *n* = 13 and *n* = 8 and (**B**) *n* = 13 and *n* = 10 for *Scn1b* WT and -null, respectively. Data represent mean ± SEM.

**Figure 7:**
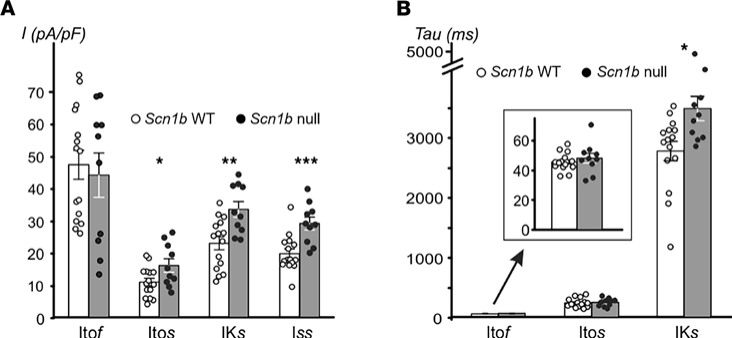
Comparison of current density, rate of decay, and availability of individual components of the potassium current. (**A and B**) Mean current density (**A**) and time constant of current decay (**B**) measured at +60 mV for I_to__f,_ I_to__s_, I_K__slow_, and I_ss_ currents in myocytes from *Scn1b* WT and -null mice. (**A**) At +60 mV current density of I_to__f_ is unchanged, whereas that of I_to__s_, I_K__slow_, and I_ss_ is increased with *Scn1b* deletion. (**B**) At +60 mV, *Scn1b* deletion results in slower decay of I_K__slow_ (inset, I_to__f_ data shown at higher magnification). *P ≤ 0.05, **P ≤ 0.01, ***P ≤ 0.001 by Student’s *t* test (assuming equal variances). (**A** and **B**) *n* = 15 and *n* = 10 for *Scn1b* WT and -null, respectively. Data represent mean ± SEM.

We recorded whole-cell potassium currents in acutely dissociated ventricular cardiac myocytes from the left ventricular wall of P17 *Scn1b* WT and -null mice to investigate the effect of *Scn1b* deletion. Whole-cell potassium currents were qualitatively similar between genotypes (Figure 5C). However, current amplitudes in myocytes from *Scn1b-*null mice were smaller compared with those from WT animals, as observed previously during examination of sodium current (10). *Scn1b-*null myocytes had a significantly smaller membrane capacitance (C_m_) (WT, C_m_ = 95.6 ± 6.1 pF, *n* = 13, and *Scn1b^–/–^,* C_m_ = 62.4 ± 5.9 pF, *n* = 8; *P* < 0.01), resulting in increased potassium current densities of approximately 24% and approximately 75% (at +70 mV) for peak current (I_peak_) and for I_end_ (Figure 6, A and B), respectively. Detailed analysis of the decay phase of the potassium current (I) revealed the presence of I_to__f_, I_to__s_, I_K__slow_, and I_ss_. With the exception of I_to__f_, the current densities (at +60 mV, from –120 mV prepulse potential) of all other components were significantly increased in *Scn1b-*null myocytes compared with WT (Figure 7A). Additionally, decay of I_K__slow_ (at +60 mV) was slowed in *Scn1b-*null myocytes (Figure 7B).

### Calcium currents are decreased in Scn1b-null ventricular cardiac myocytes.

**Figure 8:**
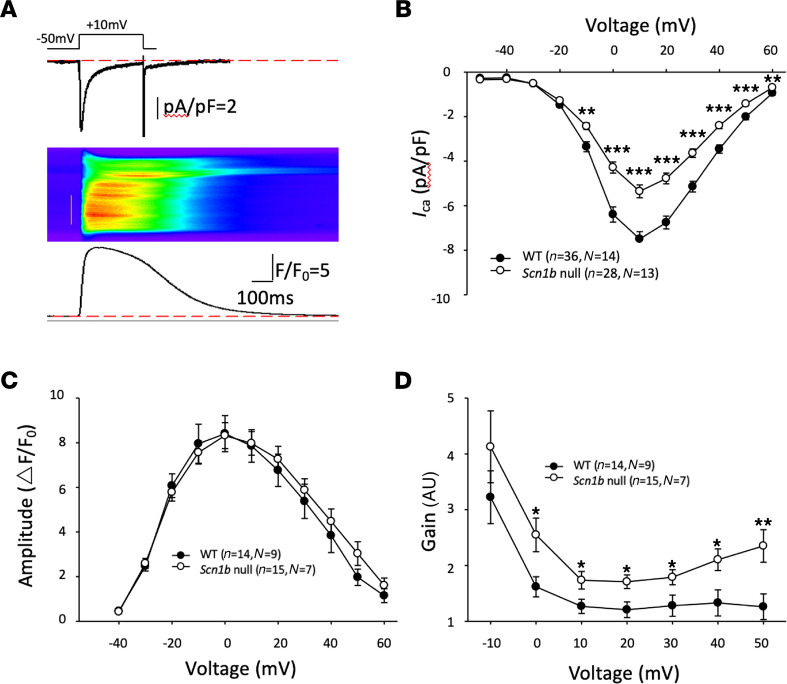
Excitation-contraction coupling in ventricular CMs from *Scn1b*-null mice. (**A**) Representative example of excitation-contraction (E-C) coupling recording. Top: *I*_Ca_ triggered by voltage clamp depolarization; middle: whole-cell Ca^2+^ transient; bottom: Ca^2+^ transient time profile. (**B**) I-V curve shows dramatically decrease of *I*_Ca_ in CMs from *Scn1b*-null mice. (**C**) Ca^2+^ transient amplitude did not change in CMs from *Scn1b*-null mice compared with WT. (**D**) E-C coupling gain (ratio between the Ca^2+^ transient amplitude and the *I*_Ca_) decreased in CMs from *Scn1b*-null mice. * *P* < 0.05; ** *P* < 0.01; *** *P* < 0.001. WT vs. *Scn1b*-null by Student’s *t* test. *N,* number of mice; *n,* number of cells. Data represent mean ± SEM.

RNA-Seq analysis of β1-ICD overexpression in CHL cells as well as of *Scn1b*-null ventricular tissue showed alterations gene expression encoding proteins known to modulate voltage-gated calcium channel activity. β1-ICD overexpression resulted in downregulated *Cacnb4* expression, encoding the calcium channel β4 subunit. In contrast, P10 *Scn1b*-null cardiac ventricle showed upregulation of the calcium channel β1 subunit gene, *Cacna1b* (Supplemental Figure 6), and P16-P17 *Scn1b*-null cardiac ventricle showed upregulation of the calcium channel β1 subunit gene*, Cacn1b*, by RT-qPCR (Supplemental Figure 4E). In general, β1-ICD overexpression led to decreased CBP gene expression, whereas *Scn1b* deletion led to increased CBP gene expression (Figure 4C). CBPs are complex regulators of voltage-gated calcium channels that can increase or decrease calcium current, depending on the particular CBP(s) at play (33). To determine whether calcium handling was altered by *Scn1b* expression in vivo, we performed whole-cell voltage clamp recording of L-type calcium current-triggered calcium transients (Figure 8A). Single ventricular cardiac myocytes were voltage clamped and depolarized from a holding potential of –50 mV to +60 mV in 10 mV increments. At the same time, intracellular calcium dynamics were imaged by confocal microscopy, using the line-scan mode, at each depolarization voltage. During the imaging, 20 mM caffeine was rapidly perfused to determine sarcoplasmic reticulum calcium content at the peak of the caffeine-elicited calcium transient. Supplemental Figure 7 shows that sarcoplasmic reticulum calcium content of cardiac myocytes was not different between genotypes. For calcium transients, the amplitude, time-to-peak, maximum rise rate, and full duration at half-maximum were analyzed. Figure 8B shows that the L-type calcium current is decreased in *Scn1b*-null mouse ventricular myocytes compared with WT. In contrast, we found no differences in the calcium transient amplitude between genotypes (Figure 8C). Finally, we calculated the excitation-contraction coupling gain (the amplification factor between calcium release from the sarcoplasmic reticulum via the ryanodine receptor and L-type calcium current), which is an indicator of intracellular calcium releasability, and found it to be increased in *Scn1b*-null myocytes compared with WT (Figure 8D).

## Discussion

VGSC β1 subunits, encoded by *SCN1B*, play important roles in cardiac physiology. *SCN1B* variants are linked to human cardiac disease, including Brugada syndrome and atrial fibrillation, although recent work suggests that *SCN1B* may not be a monogenic cause of Brugada syndrome (34). Our previous work showed that *Scn1b*-null mouse ventricular cardiac myocytes have increased transient and persistent sodium current, action potential prolongation, prolonged calcium transients, and increased incidence of delayed after depolarizations (10, 12). *Scn5a*/Nav1.5 and *Scn3a* expression, as well as ^3^H-saxitoxin binding, which measures levels of tetrodotoxin-sensitive (TTX-S) sodium channel expression, are increased in *Scn1b*-null heart (Supplemental Figure 5) (10, 12). Action potential prolongation and aberrant calcium release in *Scn1b*-null mice are TTX-S, implicating increased persistent or late sodium current via a TTX-S sodium channel α subunit, perhaps Nav1.3, leading to activation of reverse sodium/calcium exchange in the mechanism of arrhythmogenesis (12). *Scn1b*-null mouse ventricles have abnormally formed intercalated discs that show perinexal deadhesion, with significantly greater perinexal intermembrane distances compared with WT littermates, owing to the loss of β1-β1 homophilic cell adhesion (18). Finally, *Scn1b*-null mouse ECGs show prolonged QT intervals (10).

RIP substrates are involved in a wide variety of biological processes. These include, but are not limited to, neurite outgrowth, cell adhesion, lipid metabolism, receptor protein tyrosine kinase signaling, axon guidance, calcium signaling, the immune response, and cellular proliferation (28). Some of these implications may be a result of transcriptional changes downstream of RIP from substrate-ICDs. Our work suggests that the β1-ICD regulates similar gene groups. Immune response, proliferation, potassium channel, and calcium ion binding genes are upregulated in *Scn1b*-null mouse cardiac ventricle, although they are generally downregulated when the β1-ICD is overexpressed in CHL cells, suggesting that the β1-ICD may normally act as a transcriptional repressor in heart in vivo.

*Bace1*-null mice have brain region–specific, developmentally regulated alterations in *Scn1a*, *Scn2a*, and *Scn8a* expression, as well as altered sodium current and neuronal activity (35, 36). *Bace1-*null atrial cardiomyocytes have decreased steady-state potassium current (22). Cardiomyocytes isolated from transgenic mice with inducible Notch-ICD (NICD) overexpression, which is generated by RIP, have prolonged action potential duration, reduced upstroke amplitude, reduced rapidly activating voltage-gated potassium current, and reduced transient sodium current (37). Treatment of cultured neonatal mouse myocytes with a γ-secretase inhibitor to decrease NICD production resulted in increased transcript levels of *Kcnip2*, encoding the potassium channel-interacting protein 2 (KChIP2) and enhanced potassium current density (37). In other work, *Kcnip2* silencing in neonatal rat cardiac myocytes resulted in reduced levels of *Scn1b* and *Scn5*a mRNA (38). Taken together, these studies suggest that RIP substrates regulate sodium, potassium, and possibly other ion channel gene transcription. In support of this hypothesis, the data for the present study show that sodium channel β1 subunits undergo RIP through sequential intramembrane cleavage by BACE1 and γ-secretase, resulting in the generation of a soluble ICD that is translocated to the nucleus where it participates in transcriptional regulation of multiple gene families, including genes encoding sodium, potassium, and calcium channels (Summarized in the Graphical Abstract). Using an unbiased, RNA-Seq approach, we identified a subset of gene groups that are primarily downregulated when the β1-ICD is overexpressed in heterologous cells, but upregulated in *Scn1b*-null cardiac tissue, suggesting that the β1-ICD may normally act as a molecular brake on gene expression in heart in vivo. Consistent with the present RNA-Seq results, our previous work, which is repeated here, showed increased sodium current density in *Scn1b*-null ventricular myocytes compared with WT (10, 12), and new data presented here show increased potassium currents and decreased calcium currents in *Scn1b*-null myocytes compared with WT. Although β1 subunits have been shown to facilitate sodium and potassium channel α subunit targeting to the plasma membrane (39, 40), this mechanism cannot explain the increased currents recorded in *Scn1b*-null myocytes. To our knowledge, there is no evidence to date that sodium channel β1 subunits affect voltage-gated calcium channel α subunit targeting the plasma membrane. Instead, our new data suggest the β1-ICD regulates the expression of a complex group of genes encoding proteins important in modulating voltage-gated calcium channels, including calcium channel β subunits and CBPs. CBPs have been shown to both inactivate and facilitate ion conduction through the channel pore (33). The mechanism of decreased L-type calcium current observed in *Scn1b*-null ventricular myocytes is likely the result of complex gene regulation and will be the focus of future work.

Taken together, this work solidifies the critical, multifunctional roles of sodium channel β1 subunits in cardiac physiology and adds RIP-excitation coupling to the complex list of β1 functionality. Our work suggests that alterations in gene expression mediated by the β1-ICD are complex, developmentally regulated, and likely specific for cell type. Future mouse work using tissue-specific *Scn1b-*null models, inducible *Scn1b* deletion at specific developmental time points, and CRISPR knock-in of human *SCN1B* disease variants will be critical to understanding the full complexity of β1-ICD gene regulation. Identifying β1 subunit mutations that prevent RIP, as well as loss- or gain-of-function mutations that constitutively localize the β1-ICD outside or inside of the nucleus, respectively, will be vital in pinpointing exact changes in gene expression modulated via this mechanism. Finally, experiments to identify β1-ICD nuclear binding partners will be critical.

Despite the identification of a growing list of RIP substrates, the factors that initiate RIP in specific cell types and subcellular domains are poorly understood. Neuronal activity and ligand binding have been shown to activate RIP at the synapse (32), but little is known about the initiation of RIP in heart. Our previous work showed that pretreatment with γ-secretase inhibitors blocked β1-β1 trans homophilic cell adhesion–mediated neurite outgrowth (41), consistent with the idea that β1 binding to other β1 subunits on adjacent cells may initiate RIP. In ventricular myocytes, β1-β1 trans homophilic adhesion at the intercalated disk may provide a similar environment for RIP activation (18). Substrate posttranslational modification, such as ubiquitination and palmitoylation, and specific subcellular localization/co-compartmentalization have been shown to be critical factors in regulating BACE and γ-secretase cleavage (32, 42). Although our previous work has shown that β1 subunits are posttranslationally modified by glycosylation, tyrosine phosphorylation (43, 44), and palmitoylation (45), we have not yet investigated ubiquitination.

Other ion channel proteins have been shown to participate in transcriptional regulation (29, 46–49). For example, the Cav1.2 C-terminus contains a transcription factor, although the mechanism by which it is generated remains under debate. Some groups have shown that Cav1.2 encodes a transcription factor, CCAT, in its C-terminal region that is driven via a cryptic promoter located within exon 46. In contrast, others have shown the Cav1.2 C-terminus is a fragment generated by proteolysis. Regardless of its origin, evidence shows that the Cav1.2 C-terminus can localize to the nucleus and modulate transcription (46, 47, 49). Similar work on Cav1.3 demonstrated transcriptional activity of the protein’s C-terminus (48). Similar to β1, sodium channel β2 subunits are also substrates of BACE1 and γ-secretase. In neuroblastoma cells, RIP generates a β2-ICD that can translocate to the nucleus and increase *SCN1A* expression, which encodes the sodium channel α subunit, Nav1.1 (29).

Most BACE1 and γ-secretase substrate proteins are type I transmembrane proteins with extracellular domains that often contain CAM-like folds. The released C-terminal domains have been shown to translocate to the nucleus where they participate in regulating genes that are involved in cell fate determination, adhesion, migration, neurite outgrowth, axon guidance, and/or synapse formation and maintenance (28, 50). Because β1 is structurally and functionally similar to other BACE1 and γ-secretase substrates, we hypothesized that the β1-ICD generated by RIP may function in a similar manner (3, 5, 6, 11, 16, 21, 51). A large body of work has examined the transcriptional regulatory roles of the many substrate ICDs generated by BACE1 and γ-secretase (28). Notch-1, although initially cleaved by an α-secretase, is subsequently processed by γ-secretase, generating a NICD, which translocates to the nucleus to regulate transcription (52–54). The NICD associates with the DNA binding protein, CSL, and the transcriptional coactivator, Mastermind. The primary role of this assembled ternary complex is to activate transcription of Notch target genes (52). Although the Notch activator complex is well conserved, the repressor complex is more diverse and the switch between activation and repression depends on the precise cellular context during the regulatory process (55). This can be further complicated by cell type–specific effects on NICD-mediated transcriptional changes (52). The ICD generated by sequential cleavage of APP (AICD) by BACE1 and γ-secretase, AICD, forms a complex with the nuclear adaptor protein, Fe65, and the histone acetyltransferase, Tip60, to regulate transcription (56). Subsequent studies have demonstrated that the AICD can function as a transcriptional activator or as a repressor, depending on the target gene (58–61). Sodium channel β2 subunits are also substrates for intramembrane processing by BACE1 and γ-secretase (62). β2-ICD overexpression in SH-SY5Y cells increases *SCN1A* expression (29). Complex formation of the β2-ICD with other DNA binding proteins has not been investigated. Because neither the β1-ICD nor the β2-ICD contain a DNA binding domain, they may require binding partner(s) to mediate their effects on gene expression, similar to NICD and AICD.

Variants in BACE1 and/or γ-secretase substrates, as well as variants in *PSEN1*, encoding the catalytic domain of γ-secretase, are linked to many pathophysiological conditions, including Alzheimer’s disease, epileptic encephalopathy, cardiac disease, and cancer (26, 31, 44, 63–67). *SCN1B* variants, which are linked to epileptic encephalopathy and cardiac arrhythmia, may also be involved in cancer, especially through dysregulation of cell-cell or cell-matrix adhesion and transcriptional regulation. β1 Overexpression in vitro induces the growth of neurite-like projections from cultured breast cancer cells (16, 68). β1 Subunits are expressed in breast, cervical, non–small-cell lung, and prostate cancers (69), and their expression is upregulated in patient breast and prostate cancer samples (68, 70). In prostate cancer, β1 expression correlates with metastatic strength (70). β1-Overexpressing MDA-MB-231 breast cancer cells display decreased motility and proliferation compared with the parental cell line in in vitro cultures (68). Conversely, in vivo experiments using mouse xenografts of β1-overexpressing MDA-MB-231 cells resulted in promotion of primary tumor growth and metastasis compared with untransfected cells (68). Knockdown of endogenous β1 subunits in MCF-7 breast cancer cells increases cell migration (71), whereas β1 expression inhibits cell motility in cervical cancer (72). Taken together, these data suggest that the level of β1 expression modulates tumor growth and metastasis. However, it is important to note that migration is only one of many contributing factors to the invasion-metastasis cascade (73), and in vitro results examining migration are limited owing to lack of any stromal interactions (74). Discrepancies between in vivo and in vitro data may result from contributions of a heterogeneous tumor microenvironment (75). Further, it is possible that β1-mediated cell-cell adhesive interactions support apoptosis resistance, thus accounting for the increased growth rate of β1-overexpressing tumors (76). The present work suggests that transcriptional regulation via the cleaved β1-ICD may play a role in these cellular changes, and that the presence of *SCN1B* variants may affect cancer outcomes.

NICD dysregulation is similarly linked to disease. Variants in Notch receptor genes are linked to adult T cell acute lymphoblastic leukemia and lymphoma (T-LL). The most common type of Notch1 variants in human T-LL lead to ligand-independent metalloproteinase (α-secretase) cleavage (63). Activating Notch receptor variants can lead to nuclear accumulation of the NICD in T-LL. Here, nominated genes identified by sodium channel β1-ICD overexpression in CHL cells and by *Scn1b* deletion suggest that β1-ICD-mediated gene transcription may regulate proliferation, calcium ion binding, and immune response genes in vivo. Each of these gene groups has direct relationships to *SCN1B*-linked disease states, including epileptic encephalopathy, cardiac arrhythmia, and cancer (2). Thus, we propose that dysregulation of the β1-ICD signaling pathway may contribute to *SCN1B*-linked pathophysiology. In conclusion, our work adds to the multifunctionality of sodium channel β1 subunits and provides insights into disease mechanisms linked to variants in *SCN1B*. Moreover, these findings add to the growing body of evidence suggesting that substrate ICDs generated by RIP are transcriptional regulators.

## Methods

### Antibodies.

Primary antibodies used were: anti-β1_intra_ (1:1000 dilution, Cell Signaling Technologies 13950), anti-V5 (1:1000 dilution, Invitrogen, catalog R960-25), anti–α-tubulin (1:1000 dilution, Cedar Lane, CLT9002) anti–presenilin-1 (1:200, APS18 Invitrogen, catalog MA1-752), anti-BACE1 (1:1000, Invitrogen, catalog PA1-757), or anti-HSP90 (1:1000 dilution, Enzo Scientific, AC88). The specificity of anti-β1_intra_ has been shown previously by Western blot. HRP-conjugated secondary antibodies were used for Western blots in this study. Goat anti–rabbit (Invitrogen, catalog 32460) or goat anti–mouse (Invitrogen, catalog 31430) HRP-conjugated antibodies were diluted 1:1000 (anti-β1_intra_, anti–α-tubulin, anti–presenilin-1, and anti-BACE1) or 1:10,000 (anti-V5 or anti-HSP90). Alexa Fluor 568 anti-mouse was used (1:500 dilution, Invitrogen, catalog A-21043) as a secondary antibody for anti-V5 in immunocytochemistry experiments.

### Expression vectors.

A synthesis-optimized human WT β1-V5 cDNA was generated by gBLOCK from Integrated DNA technologies. The bicistronic cDNA construct included an in-frame β1 C-terminal V5 epitope tag followed by a self-cleaving 2A peptide and eGFP to facilitate immunodetection of β1 as well as transfected cells by eGFP. The eGFP alone control and β1-V5-ICD-2A-eGFP construct was generated by PCR from the respective full-length template cDNAs containing WT β1-V5. Using the Gateway cloning system, all constructs were moved from pENTR-SD/D-TOPO to pcDNAdest40 via LR Clonase reaction according to the manufacturers’ protocol.

### Cell lines.

CHL cells were originally obtained from the American Type Culture Collection (R1610, CRL-1657). HEK cells stably expressing human Nav1.5 (HEK-hNa_v_1.5 cells) were a gift from Essen BioScience. All CHL cell lines and HEK-hNa_v_1.5 cells were maintained at 37°C with 5% CO_2_ in DMEM supplemented with 5% heat-inactivated FBS and penicillin/streptomycin. Parental HEK cells were maintained at 37°C with 5% CO_2_ in DMEM supplemented with 10% heat-inactivated FBS, penicillin/streptomycin, and GlutaMAX. Stably transfected cell line media also included 600 μg/mL G418. To generate stable cell lines, 1 μg cDNAs were transfected with 5 μL Lipofectamine 2000. Forty-eight hours after transfection, cells were passed into fresh media containing 600 μg/mL G418. The cells were incubated for approximately 1 week or until eGFP-positive cell colonies were visible by epifluorescence. Individual colonies were isolated and grown until confluent and subsequently passaged for biochemical characterization. Electrophysiological experiments used transient transfection. A total of 1 µg of each cDNA was transfected with 5 μL Lipofectamine 2000. Approximately 24 hours after transfection, cells were plated onto glass cover slips for electrophysiological analysis. Electrophysiological recordings were performed approximately 24–48 hours after final plating.

### Animals.

*Scn1b* WT and -null mice were generated from mating of *Scn1b*^+/–^ mice congenic on the C57BL/6J background for over 20 *N* generations, as previously described (17). Animals were housed in the Unit for Laboratory Animal Medicine at the University of Michigan. All procedures were performed in accordance with the NIH and approved by the University of Michigan IACUC.

### Western blot analysis of cell lysates.

Cell lysates were prepared either as described below for cleavage assays or surface biotinylation assays, as appropriate. Samples were mixed with loading buffer containing SDS, 5 mM β-mercaptoethanol, and 1% dithiothreitol and heated for 10 minutes at 85°C. Proteins were separated by SDS-PAGE on 10%, 12%, or 15% polyacrylamide gels as indicated in the figure legends, transferred to nitrocellulose membrane overnight (16 hours, 55 mA, 4°C), and probed with appropriate antibodies, as indicated in the figure legends. Incubations with anti-V5 or anti-β1_intra_ and their respective secondary antibodies were performed using a SnapID with 10- to 20-minute incubations. Anti–α-tubulin and anti-HSP90 antibodies were incubated overnight at 4°C. Secondary antibodies for anti–α-tubulin and anti-HSP90 were incubated for 1 hour at room temperature (RT). Immunoreactive bands were detected using West Femto chemiluminescent substrate (GE Health Sciences) and imaged using an iBrightFL1000 system (Invitrogen). Immunoreactive signals from cleavage assays were quantified using ImageJ (NIH) and normalized to the level of α-tubulin and then to the vehicle-treated samples.

### Cleavage assays.

Stably transfected cells were grown until approximately 70% confluent in 100 mm tissue culture plates. Cells were treated with either vehicle (0.1% DMSO), varying concentrations of DAPT (Cayman Chemical) ranging from 50 nM to 1 μM, or 200 nM BSI (MilliporeSigma), as indicated in the figure legends. Twenty-four hours after treatment, cells were harvested and membranes were prepared. Briefly, harvested cell pellets were resuspended in 50 mM Tris, pH 8.0, with Complete protease inhibitors, EDTA-free (Roche). On ice, cells were broken with a Dounce homogenizer and sonicated. Lysates were spun at 2537*g* for 10 minutes to remove nuclei and other large, insoluble cell fragments. The supernatant was then removed and spun at 80,000*g* for 15 minutes at 4°C. The supernatant was removed, and the membrane-containing pellets were resuspended in 133 μL of 50 mM Tris, pH 8.0, with Complete protease inhibitors, EDTA-free (Roche), and sonicated on ice. Samples were separated on 12% SDS-PAGE gels, and Western blots were performed as described above.

### Immunocytochemistry and confocal microscopy.

CHL cells were transiently transfected with cDNA constructs, as indicated in the figure legends, with Lipofectamine 2000. Twenty-four hours after transfection, cells were fixed with ice-cold 100% methanol for 15 minutes then washed quickly 3 times with Dulbecco’s phosphate-buffered saline (DPBS). Cells were blocked for approximately 1 hour at RT in blocking buffer (90% DPBS, 10% goat serum, and 0.3% Triton X-100). Anti-V5 antibody was diluted 1:1000 in blocking buffer and incubated with cells overnight at RT in a humidified chamber. Cells were washed 3 times for 10 minutes with DPBS. Next, cells were incubated with secondary antibody for 2 hours at RT in a humidified chamber. The secondary antibody, Alexa Fluor 568, was diluted 1:500 in blocking buffer, and cells were washed 3 times for 10 minutes with DPBS and allowed to dry. Cover slips were mounted with ProLong Gold (Invitrogen) with DAPI. Transfected cells were imaged by an investigator blinded to conditions at 63x on a Zeiss 880 AiryScan confocal microscope in the University of Michigan Department of Pharmacology. Images were analyzed by an investigator blinded to condition in ImageJ using Pearson’s correlation coefficient.

### RNA-Seq.

RNA was isolated from CHL-eGFP cells, CHL-β1-ICD cells, or cardiac ventricle of P10 *Scn1b* WT or -null mice using the Qiagen RNeasy Plus kit according to the manufacturer’s instructions. Cells were lysed through a sterile, 18-gauge hypodermic needle. As fee-for-service, the University of Michigan Sequencing Core converted RNA to cDNA libraries using TrueSeq Kit (Illumina) and sequenced using Illumina HiSeq4000 with 50 cycles of paired-end sequencing. Chinese hamster reference genome, CriGri_1.0, and mouse reference genome, UCSC mm10.fa, were used as the reference genome sequences. For the ICD RNA-Seq, eGFP and β1-ICD-V5-2A-eGFP transgenes were added to the reference. Quality of reads for each sample were assessed using FastQC (version 0.11.3). The University of Michigan Bioinformatics Core Facility performed DESeq2 analysis as fee-for-service. Genes and transcripts were considered differentially expressed if they met the following 3 criteria: test status = “OK,” false discovery rate less than or equal to 0.05, and a fold change greater than or equal to 1.5.

### RT-qPCR.

RNA was isolated from cardiac ventricles of P10 or P16-17 *Scn1b* WT or -null mice or from CHL cells, as indicated in the figure legends, using the Qiagen RNeasy Plus kit according to the manufacturer’s instructions. Cell or tissues were lysed through a sterile, 18-gauge hypodermic needle or vortexed for 30 seconds (heterologous cells). RNA was stored at –80°C until use. cDNA was generated from 1–2 μg of RNA using Reverse Transcriptase SuperScript III (RT SS III) and random primers (Invitrogen). Primers, dNTPs, and RNA were incubated at 65°C for 5 minutes. Salt buffers, RT SS III, and RNaseOUT were added and incubated at 25°C for 5 minutes, 50°C for 60 minutes, and then at 75°C for 15 minutes. cDNA was diluted 1:3- to 1:5-fold in water. Comparative qPCR using SYBR Green (Applied Biosystems) and gene-specific primers (Integrated DNA Technologies) was performed. ΔΔCt values were calculated by comparing genes of interest with GAPDH and normalizing to the control condition (WT or lipofectamine-only treatment) to determine comparative gene expression. Data are presented as gene expression ± SEM. Statistical significance (*P* value less than 0.05) of comparisons between genotypes was determined using Student’s *t* test. Statistical significance (*P* value less than 0.05) of comparisons between lipofectamine-treated, eGFP, and WT β1-ICD-V5 transfected cells was determined using 1-way ANOVA for each examined gene.

### Measurement of sodium currents by whole-cell voltage clamp.

Sodium current recordings from acutely dissociated mouse myocytes were performed as previously described (10). Voltage clamp recordings were performed on heterologous cells at RT in the whole-cell configuration using a Multiclamp 700B amplifier and pClamp (versions 11, Molecular Devices) with 1.5–2.5 MΩ patch pipettes. Sodium currents were recorded in the presence of a bath solution containing (in mM): 120 NaCl, 1 BaCl_2_, 2 MgCl_2_, 0.2 CdCl_2_, 1 CaCl_2_, 10 HEPES, 20 TEA-Cl, 10 glucose (pH 7.35 with CsOH, Osmolarity: 300–305 mOsm). Fire-polished patch pipettes were filled with an internal solution containing (in mM): 1 NaCl, 150 N-methyl-D-glucamine, 10 EGTA, 2 MgCl_2_, 40 HEPES, 25 phosphocreatine-Tris, 2 MgATP, 0.02 Na_2_GTP, 0.1 Leupectin (pH 7.2 with H_2_SO_4_). Sodium current was recorded in response to a series of voltage steps between –100 and +30 mV in 5 mV increments, from a holding potential of –90 mV for 200 milliseconds. A step back to –20 mV for 200 milliseconds was used to determine the voltage dependence of inactivation. Series resistance was compensated 40%–65%, and leak subtraction performed by application of a standard P/4 protocol. Normalized conductance and inactivation curves were generated as described previously (31). Current densities were determined by dividing current amplitude by the cell capacitance (Cm), as determined by application of +10 mV depolarizing test pulses. For ICD peptide experiments, 200 μM peptide was used.

### Measurement of potassium currents in mouse cardiac myocytes.

Ventricular cardiac myocytes were acutely isolated from P16-P19 *Scn1b* WT or -null mice as previously described (77). The bath solution contained in mM: 137 NaCl, 5.4 KCl, 1.5 CaCl_2_, 0.5 MgCl_2_, 10 HEPES, 0.16 NaH_2_PO_4_, 3 NaHCO_3_, 0.002 nicardipine, 0.02 ouabain, pH 7.35, with NaOH. Nicardipine and ouabain were used to block L-type calcium channels and Na/K pumps, respectively. Stock solutions for nicardipine (10 mM) and ouabain (20 mM) were prepared in DMSO and H_2_O, respectively, and diluted to the appropriate concentration in bath solution before use. Patch pipettes (2–3 MΩ) were filled with (in mM): 130 KCl, 2 K_2_-ATP, 1 EGTA, 10 HEPES, pH 7.3, with KOH. Series resistance was routinely compensated to approximately 80% before the recordings. Holding potential was set to –70 mV and current traces filtered at 1 kHz. To assess voltage dependence of activation, whole-cell outward potassium currents were recorded in response to 5second depolarizing voltage steps to potentials between –70 and +70 mV from a 5-second –120 mV prepulse potential in 10 mV increments at 15-second intersweep intervals. The values of the I_peak_ and I_end_ (end-current) were obtained at approximately 20 milliseconds (variable) and 4.88 seconds after the beginning of the depolarization, respectively. The decay phases of the outward potassium currents were fit by the sum of 3 exponentials using the following expression: I(t) = I_to__f_ × e^–t/τf^ + I_to__s_ × e^–t/τs^ + I_K__slow_ × e^–t/τK^ ^slow^ + I_SS_, where t is time, τ_f_, τ_s_, and τ_K__slow_ are the time constants of decay of I_to_ _fast_ (I_to__f_)_,_ I_to_ _slow_ (I_to__s_), and I_K__slow_. I_ss_ denotes the steady-state current. In practice, to improve the fit (to account for vast differences in the time constants), τ_f_ was determined first using 2-exponential approximation over a reduced time span, and then 3-exponential fit over the entire time span was performed with known (fixed) τ_f_. The amplitudes of individual components of K currents were recalculated to zero time (beginning of the depolarizing pulse) using corresponding time constants.

### Measurements of calcium currents, calcium transients, and sarcoplasmic reticulum calcium content in mouse cardiac myocytes.

Ventricular cardiac myocytes were acutely isolated from P16-P19 *Scn1b* WT or -null mice as previously described (77). Calcium current and calcium current-triggered whole-cell calcium transients were recorded simultaneously as previously described (77). Briefly, single ventricular myocytes were depolarized from a holding potential of –50 mV to +60 mV in 10 mV increments for 300 milliseconds. At the same time, intracellular calcium dynamics were imaged by confocal microscopy using the line-scan mode of a Nikon A1R microscope at each depolarization voltage. Sarcoplasmic reticulum calcium content was measured as previously described (78). Briefly, single ventricular myocytes were loaded with fluo-4-AM (Thermo Fisher Scientific) and imaged by confocal microscopy in line-scan mode. A total of 20 mM caffeine was rapidly perfused onto the cell and sarcoplasmic reticulum calcium content was determined by the peak of the caffeine-elicited calcium transient.

### Data availability.

RNA-Seq data have been submitted to the repository at NCBI GEO (accession numbers GSE136927 and GSE136535; https://www.ncbi.nlm.nih.gov/geo/query/acc.cgi?acc=GSE136927 and https://www.ncbi.nlm.nih.gov/geo/query/acc.cgi?acc=GSE136535, respectively)

### Statistics.

Statistical analyses for cleavage assay experiments were performed with *n* = 3–4 for each experiment. The DAPT concentration response and γ-secretase inhibitor experiments were 1-way ANOVA with multiple comparisons. Data are represented as mean ± SEM. All Student’s *t* tests were 2 tailed. β1 Mutant cleavage experiments were performed as unpaired 2-tailed Student’s *t* tests between vehicle- and DAPT-treated groups. Sodium current recordings had an *n* of 10–15 cells per condition for each heterologous expression experiment from a minimum of 3 independent transfections or an *n* of 10 cells from a total of 3 mice from each genotype for acutely dissociated myocytes. The voltage dependence of activation and inactivation were compared using nonlinear fit, maximum current was analyzed using 1-way ANOVA with multiple comparisons, and current density was compared with the control, eGFP, with an unpaired Student’s *t* test at each voltage step. Statistical analysis of potassium current data (*n* = 8–15 per condition) was performed using Student’s *t* test (assuming equal variances). Statistical analysis of calcium current data (*n* = 13–15 per condition) was performed using Student’s *t* test (assuming equal variances). Sarcoplasmic reticulum data (*n* = 31–38 per condition) were compared using Student’s *t* test. Analysis of colocalization between the β1-ICD constructs and nuclei (DAPI) was performed blinded to condition using the ImageJ coloc2 package. Pearson’s correlation coefficient for each cell (*n* = 11–17 for each condition, from 3 independent transfections) was recorded. One-way ANOVA with multiple comparisons was performed. Data are represented as mean ± SD. Differences were considered significant if the *P* value was less than 0.05. The University of Michigan Bioinformatics Core Facility performed DESeq2 analysis as a fee-for-service. In each experiment, *n* = 4 was used for each condition. Genes and transcripts were considered differentially expressed if they met the following 3 criteria: test status = “OK,” false discovery rate less than or equal to0.05, and a fold change greater than or equal to 1.5.

### Study approval.

This study was approved by the University of Michigan IACUC under protocol PRO00008784.

## Author contributions

AAB performed or contributed to all cloning, generated all stable cell lines, and performed cleavage assays, imaging, biotinylations, and RNA isolations for RNA-Seq, RT-qPCR, and transfections for electrophysiology. SLH performed RNA isolations and RT-qPCR experiments for the P16-P17 mouse cohort. NE and LFLS performed sodium current recordings and analyses. ANL and LP performed potassium current recordings and analyses. YTZ performed calcium current recordings and analyses. AMP and JO contributed to cloning of cDNA constructs. AMP performed colocalization analyses. JO contributed to experimental design. LLI contributed to experiment design and interpretation. AAB and LLI cowrote the manuscript.

## Supplementary Material

Supplemental data

## Version 1 01/07/2021

In-Press Preview

## Version 2 02/08/2021

Electronic publication
