# Supplementary material for: Sodium channel β1 subunits participate in regulated intramembrane proteolysis-excitation coupling
Source: JCI Insight. 2021 Feb 8;6(3):e141776. doi: 10.1172/jci.insight.141776 (PMC7934843; doi:10.1172/jci.insight.141776)
Supplement: Supplemental data [file jciinsight-6-141776-s244.pdf]

Supplemental Data

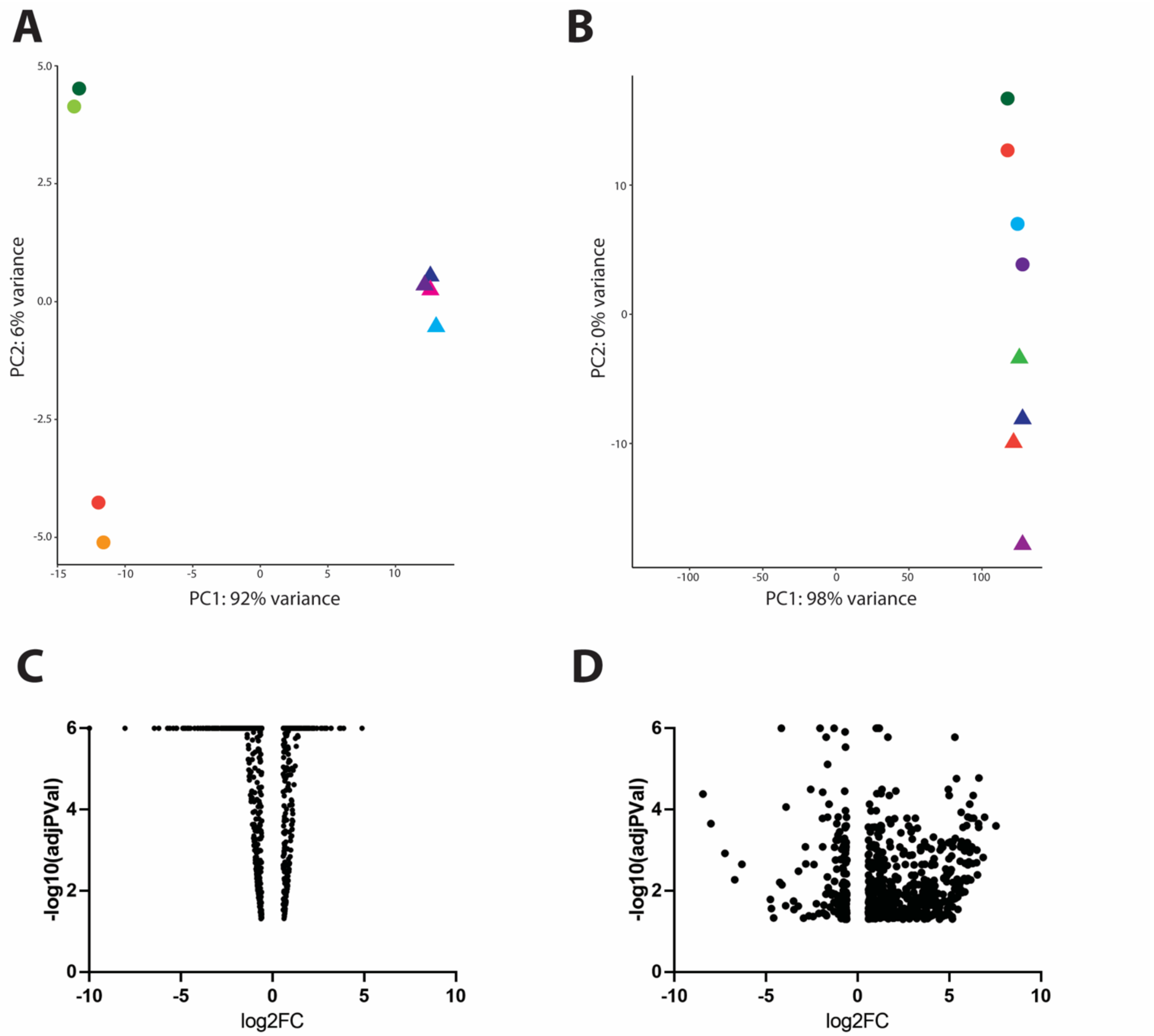

Supplemental Figure 1. Principal Component Analysis (PCA) and Volcano plots for RNA-Seq experiments. Volcano plots display only data points with a fold-change of 1.5 or greater and an adjusted p-value of less tha 0.05. **A.** CHL cells stably overexpressing eGFP only (▲) or β1-ICD-V5-2A-eGFP (●) samples bin according to genotype. **B.** *Scn1b* WT (▲) and *Scn1b* null (●) P10 mouse cardiac ventricle bin according to genotype. **C.** RNA-Seq volcano plot for CHL cells stably overexpressing eGFP only or β1-ICD-V5-2A-eGFP. **D.** RNA-Seq volcano plot for P10 WT and *Scn1b* null cardiac ventricle.

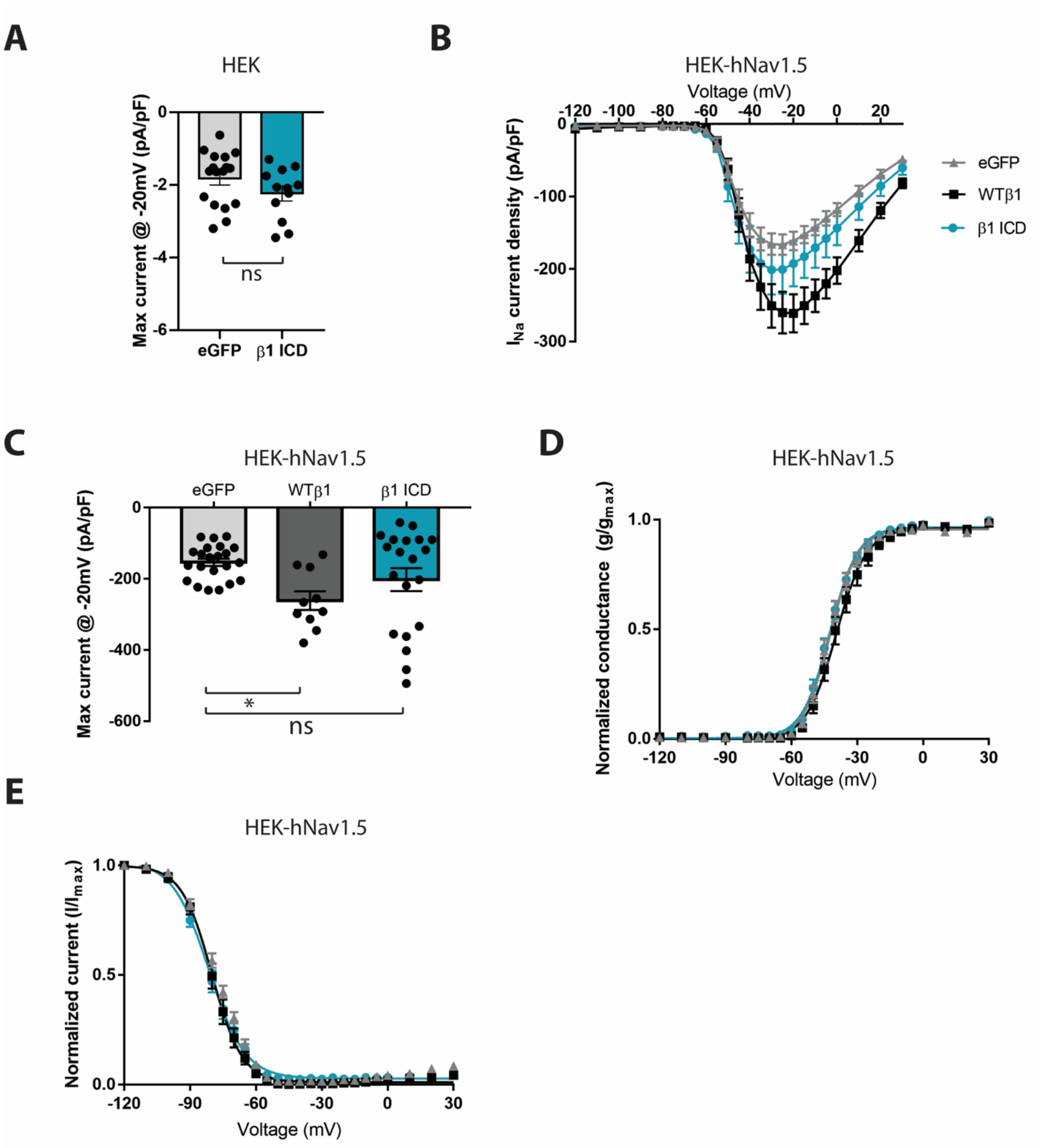

Supplemental Figure 2. β1-ICD has no effect on sodium current in heterologous cells.
A. Expression of β1-ICD-V5-2Ae-GFP does not increase sodium current density in HEK cells compared to control, eGFP-only. N=12-17 per condition. **B.** Sodium current density is increased when WT β1-V5-2A-eGFP is co-expressed with $hNa_v1.5$ compared to eGFP-only control. Co-expression with β1-ICD-V5-2A-eGFP does not increase sodium current density compared to eGFP-only control. **C.** Maximal current is increased when WT β1-V5-2A-eGFP, but not β1-ICD-V5-2A-eGFP, is co-expressed with $hNa_v1.5$ compared to eGFP control. **D.** Co-expression of WT β1-V5-2A-eGFP or β1-ICD-V5-2A-eGFP has no effect on the voltage-dependence of sodium current activation compared to eGFP control. **E.** Co-expression of WT β1-V5-2A-eGFP or β1-ICD-V5-2A-eGFP has no effect on the voltage-dependence of sodium current inactivation compared to eGFP control. N=10-24 per condition. The voltage-dependence of activation and inactivation

were compared using nonlinear fit. Max current was compared using Student's t-test or one-way ANOVA. Data are represented as the mean ± SEM.

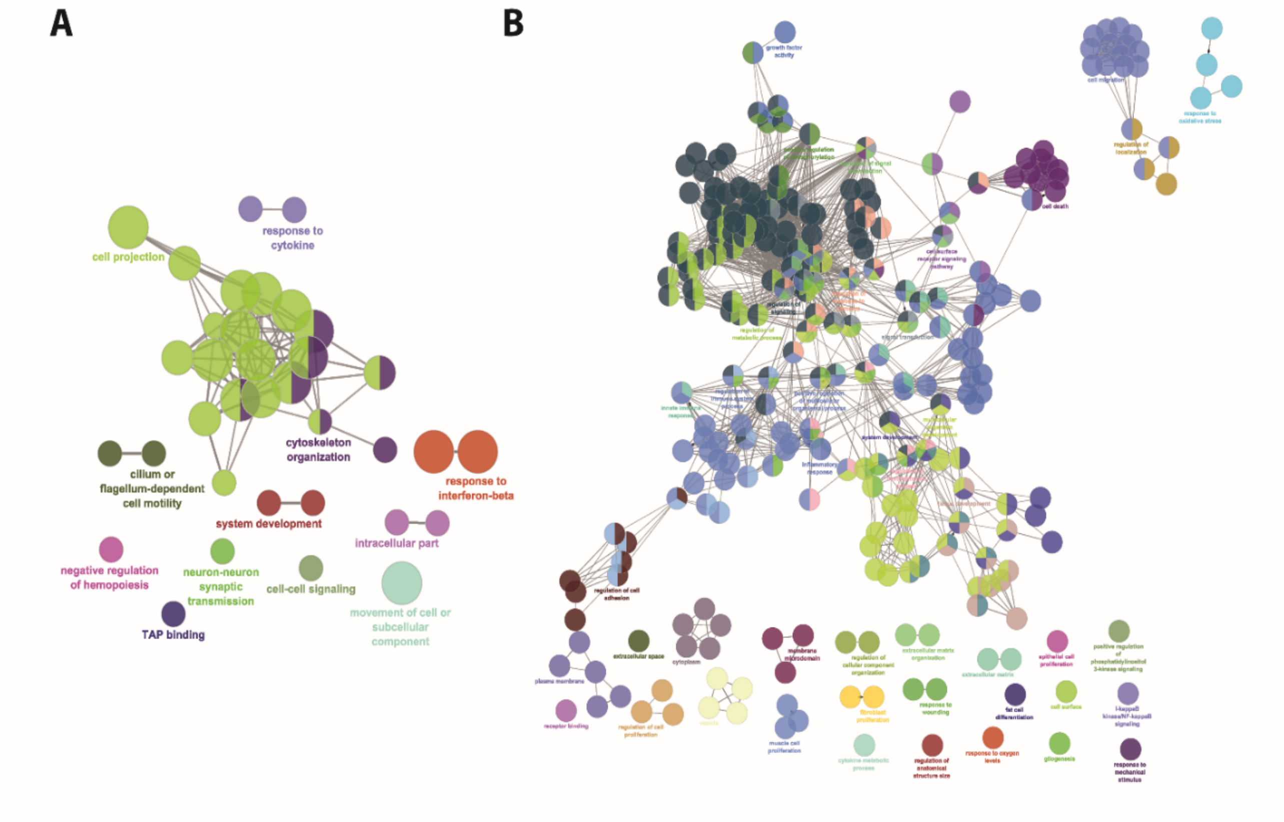

Supplemental Figure 3. ClueGO network analysis of RNA-Seq data sets. **A.** ClueGO network analysis for *Scn1b* WT vs. *Scn1b* null cardiac ventricle RNA-seq for GO groups with an adjusted p-value ≤ 0.05. **B.** ClueGO network analysis for CHL cells stably overexpressing eGFP or the β1-ICD and eGFP for GO groups with an adjusted p-value ≤ 0.0001.

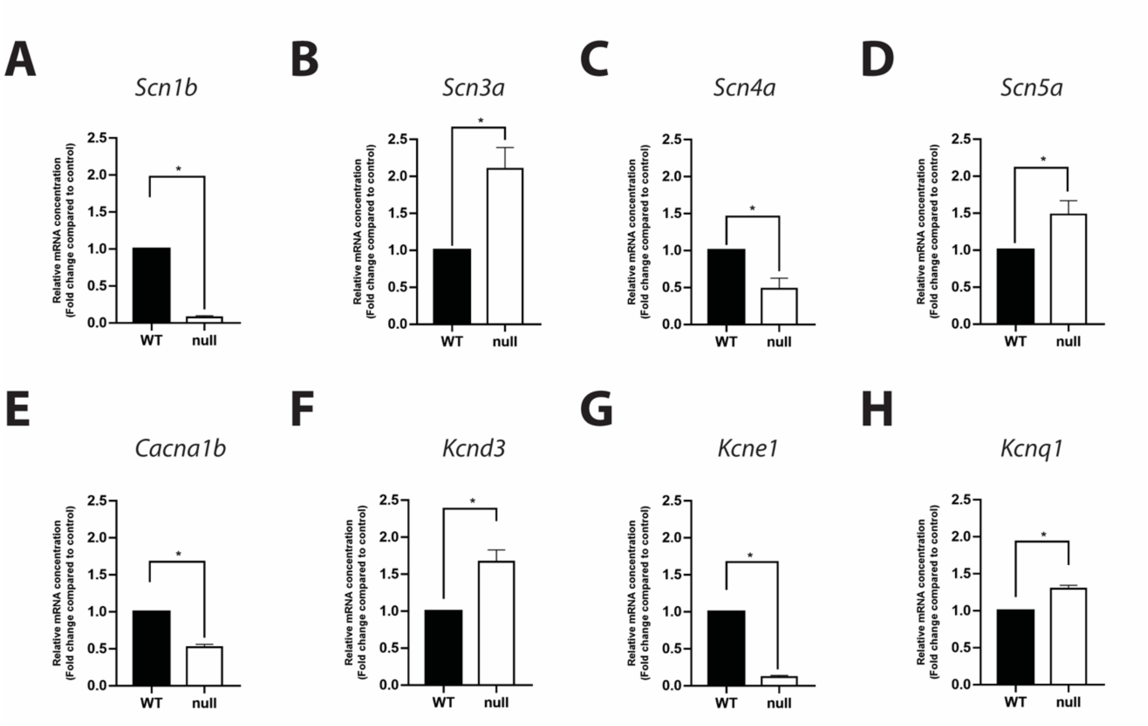

Supplemental Figure 4: RT-qPCR analysis of ion channel genes from P16-P17 WT and *Scn1b* null cardiac ventricle. Statistical significance was determined using Student's T-test (p-value < 0.05). **A.** Relative expression of *Scn1b* (confirmation of *Scn1b* deletion). **B.** Relative expression of *Scn3a*. **C.** Relative expression of *Scn4a.* **D.** Relative expression of *Scn5a.* **E.** Relative expression of *Cacna1b.* **F.** Relative expression of *Kcnd3*. **F.** Relative expression of *Kcne1*. **H.** Relative expression for *Kcnq1.* Data are represented as the mean ± SEM.

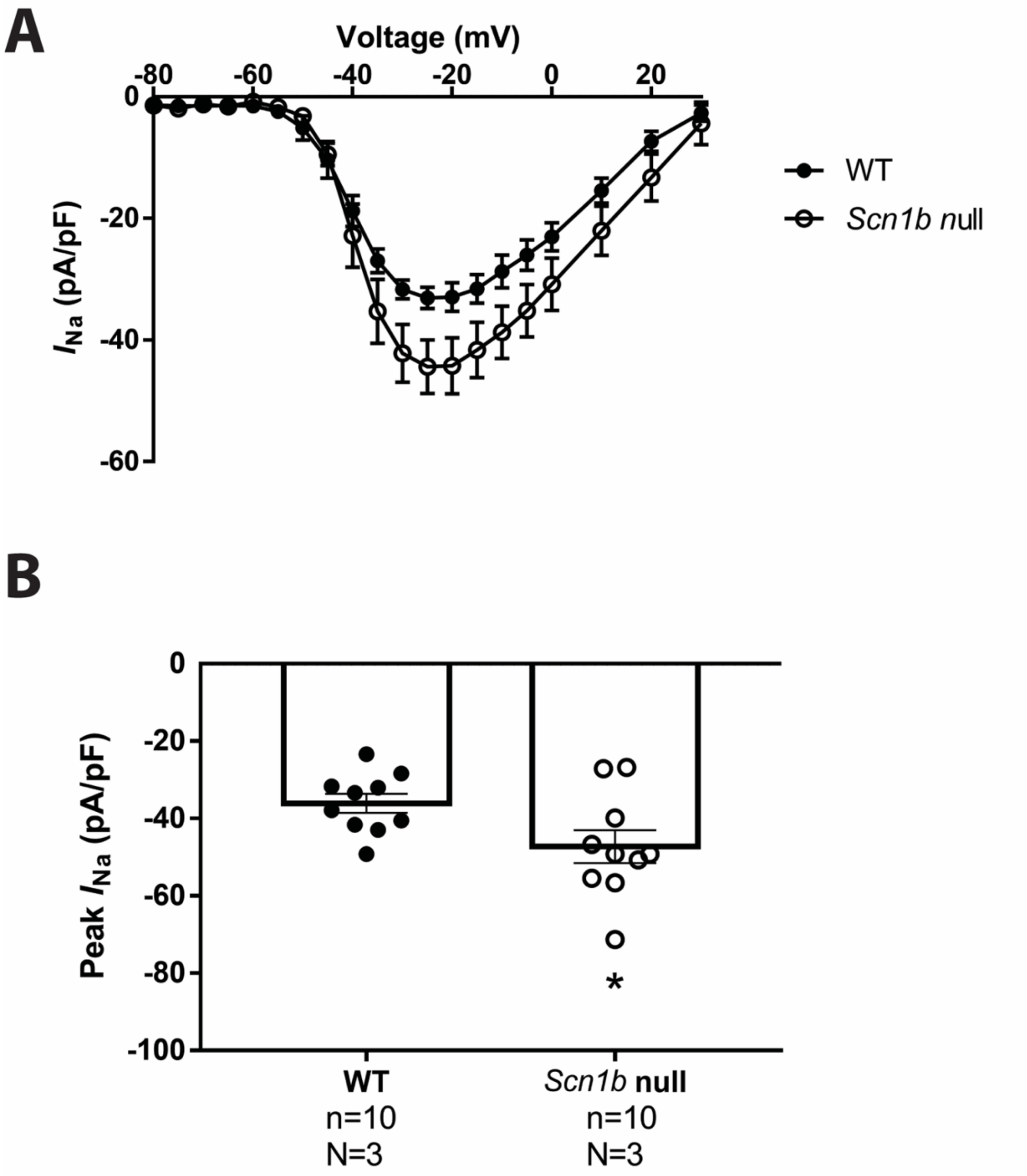

Supplemental Figure 5: Sodium current density is increased in acutely isolated P16-17 *Scn1b* null cardiac ventricular myocytes (open circles) compared to WT (closed circles). **A.** Current-voltage relationship. **B.** Maximal current density (pA/pF). The voltage-dependence of activation and inactivation were compared using nonlinear fit. Max current was compared using Student's t-test or one-way ANOVA. Data are represented as the mean ± SEM.

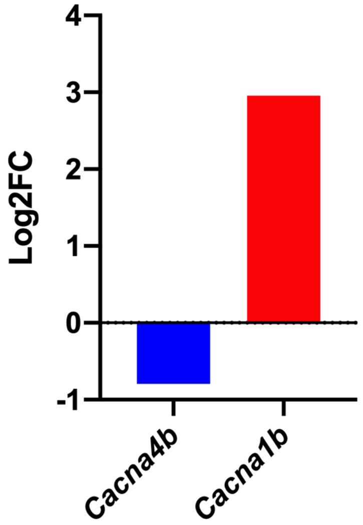

Supplemental Figure 6. RNA-Seq analysis showing changes in voltage-gated calcium channel β subunit genes. Blue bar: ICD overexpression experiment. Red bar: *Scn1b* deletion. N=4.

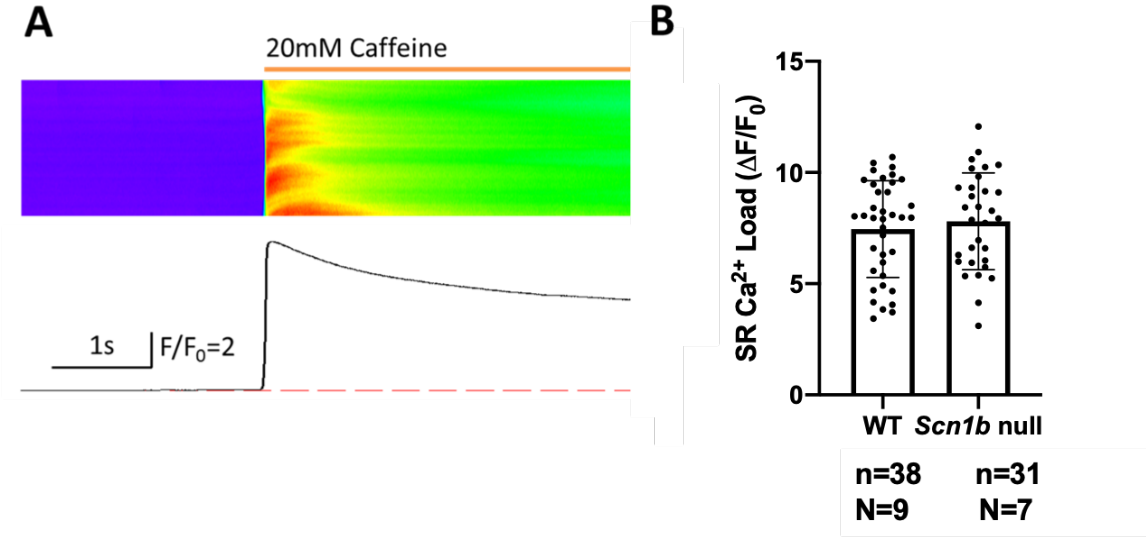

Supplemental Figure 7. Sarcoplasmic reticulum (SR) Ca^{2+} content is similar in acutely isolated P16-17 *Scn1b* null and WT ventricular cardiac myocytes. **A**. Representative example of SR Ca^{2+} content measurement. **B**. Student's t-test showed no difference in SR Ca^{2+} content between genotypes. N: number of mice; n: number of cells. Data are represented as the mean ± SEM.

	Transient I_{Na} density (pA/pF)	Persistent I_{Na} density (pA/pF)
**hNa$_v$1.5 alone**	-156.8±30.2	-0.9±0.2
**hNa$_v$1.5+ICD peptide**	-177.6±29.5	-0.6±0.2

Supplemental Table 1. Application of β1-ICD peptide through the patch pipet had no effect on hNav1.5-generated transient or persistent sodium current (I_{Na}) density. Statistical significance was determined using student's t-test. N=8-10 cells per condition.

	Voltage dependence						
	**Activation**			**Inactivation**			**n**
	**G_{max} (nS)**	***k (mV)***	**$V_{1/2}$ (mV)**	***h* (mV)**	**$V_{1/2}$ (mV)**	**C**	
**hNa$_v$1.5**	1.95±0.42	6.56±0.47	-42.7±1.6	-9.25±0.42	-88.7±4.6	0.02±0.005	8
**hNa$_v$1.5+ICD peptide**	2.16±0.32	5.87±0.31	-44.2±1.1	-9.05±0.35	-85.9±2.2	0.02±0.003	10

Supplemental Table 2. Application of the β1-ICD peptide through the patch pipet had no effect on the voltage-dependence of activation or inactivation of hNa$_v$1.5-generated sodium current. The voltage-dependence of activation and inactivation were compared using nonlinear fits. N=8-10 cells p
